# A Comparative Study of Infusion of Ephedrine and Phenylephrine on Hemodynamic Stability After Spinal Anesthesia in Elderly Patients Undergoing Lower Limb Orthopedic Surgeries

**DOI:** 10.7759/cureus.69977

**Published:** 2024-09-23

**Authors:** Syed Sufian Ibrahim, Basavaraj Patil

**Affiliations:** 1 Anesthesiology, Shri BM Patil Medical College, Hospital and Research Centre, BLDE (Deemed to be University), Vijayapura, IND

**Keywords:** elderly, ephedrine, hypotension, phenylephrine, spinal anesthesia

## Abstract

Background

Hypotension during spinal anesthesia occurs due to sympathetic nervous system blockade, resulting in decreased systemic vascular resistance and reduced cardiac output. Due to reduced sympathetic tone, peripheral arterial vasodilatation and blood pooling in lower limbs due to venodilatation occur, reducing preload to the heart and stroke volume. The elderly patients have reduced cardiovascular compensatory mechanisms, increasing the frequency and severity of hypotension due to sympathetic blockade after spinal anesthesia significantly. Vasopressors correct hypotension during the intraoperative period to maintain organ perfusion. Fluids can be administered, and if given excessively, can cause fluid overload and urinary retention. This study aimed to compare the effectiveness of vasopressors, phenylephrine, and ephedrine in maintaining hemodynamic stability intraoperatively through prophylactic infusion after spinal anesthesia in elderly patients for lower limb orthopedic surgeries.

Methodology

A total of 174 elderly patients aged 60 years and above with American Society of Anesthesiologists (ASA) Classification I and II, undergoing lower limb orthopedic surgeries, enrolled in a randomized comparative study, were allocated into three groups: Group P (phenylephrine, n=58) received 250 mcg phenylephrine in 30 ml normal saline using infusion syringe pump, Group E (ephedrine, n=58) received 30 mg ephedrine in 30 ml normal saline using infusion syringe pump, and Group C (control group, n=58) received mephentermine I/V (6 mg bolus) when the fall in blood pressure was below 30% of baseline without any placebo infusion. Hemodynamic parameters (systolic and diastolic blood pressure, mean arterial pressure (MAP), heart rate) at 15, 10, and 5-minute intervals before spinal anesthesia, and at 3, 6, 9, 12, 15, 20, 25, and 30-minute intervals after spinal anesthesia. The need for rescue doses to treat hypotension after spinal anesthesia was recorded.

Result

At all time intervals following spinal anesthesia, Group E reported heart rate and systolic blood pressure better than Groups P and C, significantly. At 3, 6, 9, 12, 15, and 25-minute intervals following spinal anesthesia, the diastolic blood pressure in Group E was enhanced significantly than Groups P and C. The MAP in Group E was substantially higher than in other groups at 3, 6, 15, and 20-minute intervals following spinal anesthesia, which was statistically significant. Compared to Groups P and C, Group E required lesser rescue doses to treat intraoperative fall in hypotension 30% below baseline and lesser events of bradycardia.

Conclusion

Following spinal anesthesia, the preload to the heart is to be maintained with intravenous (crystalloid or colloidal) solutions to maintain cardiac output adequately. Intraoperative use of phenylephrine and ephedrine as a low-dose prophylactic infusion can be used, as it increases both systemic vascular resistance and preload without cardiac stimulation along with intravenous solutions to maintain hemodynamic parameters such as systolic and diastolic blood pressure, MAP, heart rate effectively but preferably ephedrine in elderly patients.

## Introduction

Hypotension is frequently observed after induction of spinal anesthesia which is a commonly used regional technique in elderly patients undergoing lower limb orthopedic procedures [1]. It occurs due to reduced cardiac output and peripheral vascular resistance, which raises the possibility of myocardial ischemia. Impaired cerebral auto-regulation during the perioperative period decreases middle cerebral artery velocity [2]. Reducing the spinal dose and the extent of anesthetic spread to the necessary dermatomes decreases the incidence of adverse effects [3]. As crystalloids are frequently ineffective in ameliorating blood pressure, their administration can soon result in circulatory overload and congestive cardiac failure [4].

The elderly are more likely to have comorbid diseases, which increases their risk of hypoperfusion, which is due to hypotension, the primary risk factor for which is hypovolemia [4,5]. Vasopressors such as phenylephrine and ephedrine can be immediately infused into elderly people to prevent hemodynamic abnormalities following spinal anesthesia [6]. More research is to be done to study the efficacy and minimal dose requirement of vasopressors as a prophylactic infusion to maintain hemodynamics in the elderly population.

The objective of this study was to determine the effectiveness of vasopressors such as phenylephrine and ephedrine as a low-dose prophylactic intravenous infusion to study hemodynamic variations in elderly patients immediately after spinal anesthesia and to prevent hypotension and the need for rescue doses to maintain vital parameters in patients aged 60 years and above undergoing lower limb orthopedic surgeries.

## Materials and methods

This study was registered at Clinical Trials Registry-India with link https://ctri.nic.in/Clinicaltrials/login.php under the CTRI/2023/04/051451 registration number. The study time was from May 2023 to August 2024. The study was conducted after receiving approval from the institutional ethical committee of Bijapur Lingayat District Education (BLDE) (Deemed to be University), Shri BM Patil Medical College, Hospital and Research Centre with ethical clearance number BLDE(DU)/IEC/787/2022-23. A total of 174 patients aged 60 years and above belonging to American Society of Anesthesiologists (ASA) grades I and II [7], scheduled for elective lower limb orthopedic surgical procedures under spinal anesthesia, were included in the study. The study excluded patients with infection at the site of injection, uncontrolled hypotension, allergic or hypersensitivity to vasoconstrictors, coagulopathies, sepsis, fixed cardiac output states, uncontrolled hypertension, hypothyroidism, uncontrolled diabetes, untreated or uncontrolled heart failure, pheochromocytoma, on monoamine oxidase inhibitors, tricyclic antidepressants, phenothiazine compounds, recent myocardial ischemia/infarction.

During the pre-anesthetic evaluation, a detailed history and general and systemic examinations were carried out the previous day. A history of any significant medical illness was elicited and medication history was taken. The airway, respiratory system, and cardiovascular system were assessed. Written informed consent was obtained. Routine investigations such as complete blood profile, bleeding time and clotting time, random blood sugar, serological tests, chest X-ray, and 2D echocardiography were performed.

On the day of the surgery, nil per oral status was confirmed. An 18G IV cannula was secured and preloading with Ringer lactate solution (15 ml/kg) was infused. On the operating table, vital monitors such as non-invasive blood pressure (NIBP), electrocardiography (ECG), and pulse oximetry were attached to record baseline heart rate, blood pressure, and SpO2. The patients were positioned in a sitting posture. Using a 25G Quincke spinal needle, a lumbar puncture was done at the L3-4 or L4-L5 interface following skin disinfection and 2% lignocaine infiltration. Premedication was given with midazolam 0.5 mg I/V to alleviate the anxiety of the patients. The groups were intrathecally administered with 0.5% hyperbaric bupivacaine 15 mg I/V. Subsequently, patients were supined and oxygen (5 L/min) was delivered with a face mask. Five minutes after intrathecal injection, sensory blockade of spinal anesthesia was assessed using ice cubes or pinpricks.

Patients were split randomly into three groups. Group P received a low-dose continuous infusion of 30 ml 0.9% normal saline with 250 mcg of phenylephrine for 30 minutes (8.3 mcg/min) immediately following spinal anesthesia using an infusion syringe pump. Group E received a continuous infusion of 30 ml 0.9% normal saline with 30 mg of low-dose ephedrine for 30 minutes (1 mg/min) immediately following spinal anesthesia using an infusion syringe pump. Group C (control) received a bolus of mephentermine 6 mg I/V after spinal anesthesia when required without any placebo infusion. The infusion of treatment medication in Group P and Group E was started immediately after spinal anesthesia at an infusion rate of 60 ml/hour, and infusion was stopped after 30 mins (study period) following induction of spinal anesthesia. NIBP, heart rate, ECG, and percentage of oxygen saturation (SpO2) were monitored. Incidence of hypotension and bradycardia were noted. Hemodynamic parameters were recorded at 15, 10, and 5 minutes prior to spinal anesthesia and for 0, 3, 6, 9, 12, 15, 20, 25 and 30-minute intervals after spinal anesthesia.

The rescue treatment protocol to treat intraoperative hypotension was followed. If the reduction in systolic blood pressure was greater than 30% below baseline, then a bolus dose of phenylephrine 50 mcg I/V or ephedrine 5 mg I/V was given in respective groups. Atropine 0.6 mg I/V was given if the heart rate was less than 50 beats per minute. In the case of hypertension, the ongoing infusion was stopped.

Statistical analysis

Using G*Power Version 3.1.9.4 (Heinrich-Heine-Universitat Dusseldorf, Dusseldorf, Germany) for sample size calculation, the mean arterial pressure (MAP) for control (mean=90, SD=12), phenylephrine (mean=85, SD=12), and ephedrine (mean=84, SD=9) were obtained. This study required a total sample size of 174 (for each group 58, assuming equal group sizes) to achieve a power of 80% for detecting a difference means [7].

The data was entered into a Microsoft Excel sheet and statistical analysis was performed using IBM SPSS Statistics for Windows, Version 20 (IBM Corporation, New York, US). The findings were evaluated using basic statistics, including mean and standard deviation of numerical variables and percentages for categorical variables. To compare categorical variables between the two groups, the chi-square test was used. For groups that were not normally distributed, the Kruskal-Wallis test was performed. All analyses were two-tailed, with a p-value of <0.05, which was considered to be statistically significant.

## Results

**Figure 1:**
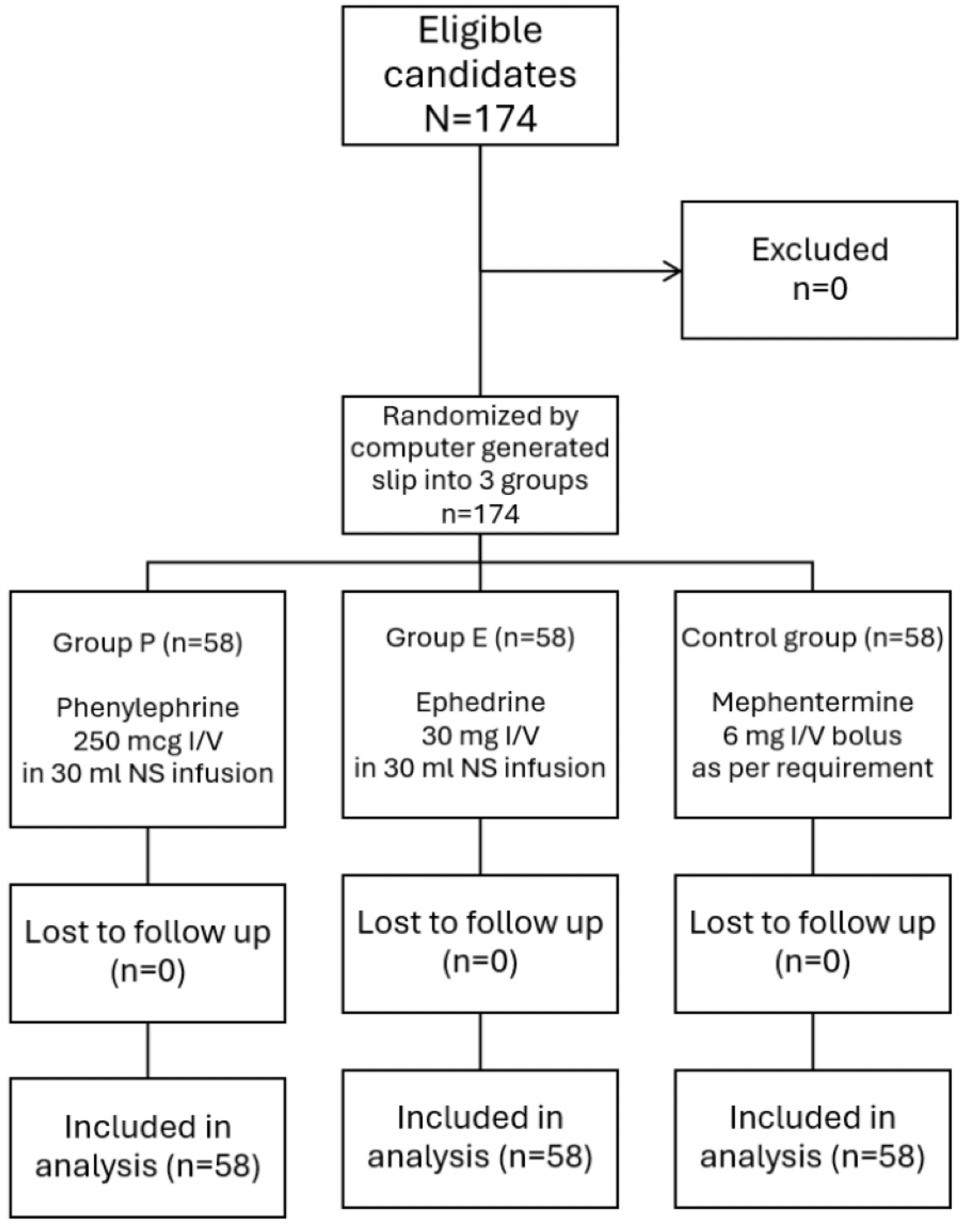
CONSORT flow diagram CONSORT: Consolidated Standards of Reporting Trials; n: Number of patients

In the present study, constituting 174 elderly patients aged 60 years and above, 58 in each group were posted for all elective lower limb orthopedic surgeries (such as open reduction and internal fixation of fractures, tendon repairs, soft tissue procedures, arthroplasty, arthroscopic knee surgeries, etc.) to assess the impact of phenylephrine or ephedrine infusion administered intravenously as a prophylactic measure to prevent hypotension and its effect on heart rate. The present study followed The Consolidated Standards of Reporting Trials (CONSORT) guidelines (Figure 1). There was no statistically significant difference in all groups with demographic characteristics such as age (p=0.496), sex (p=1), ASA grades (p=0.197), and body mass index (p=0.271).

**Figure 2:**
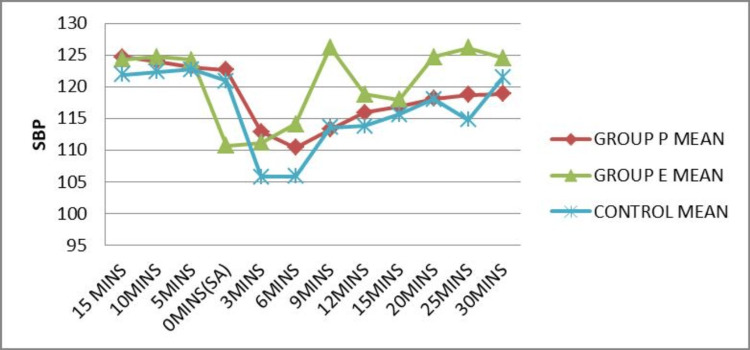
Comparison of SBP before and after SA SBP: Systolic blood pressure; SA: Spinal anesthesia

The mean systolic blood pressure at different time intervals was plotted for each group (Figure 2). The mean systolic blood pressure at induction (0 mins) of spinal anesthesia was superior in Group P compared to Group E and the control group. It was enhanced in Group E when compared to other groups at 6 (p=0.001), 9 (p=0.001), 12 (p=0.01), 15 (p=0.02), 20 (p=0.001), 25 (p=0.001) and 30 (p=0.002) minute time intervals after spinal anesthesia, which was statistically significant (p<0.05).

**Figure 3:**
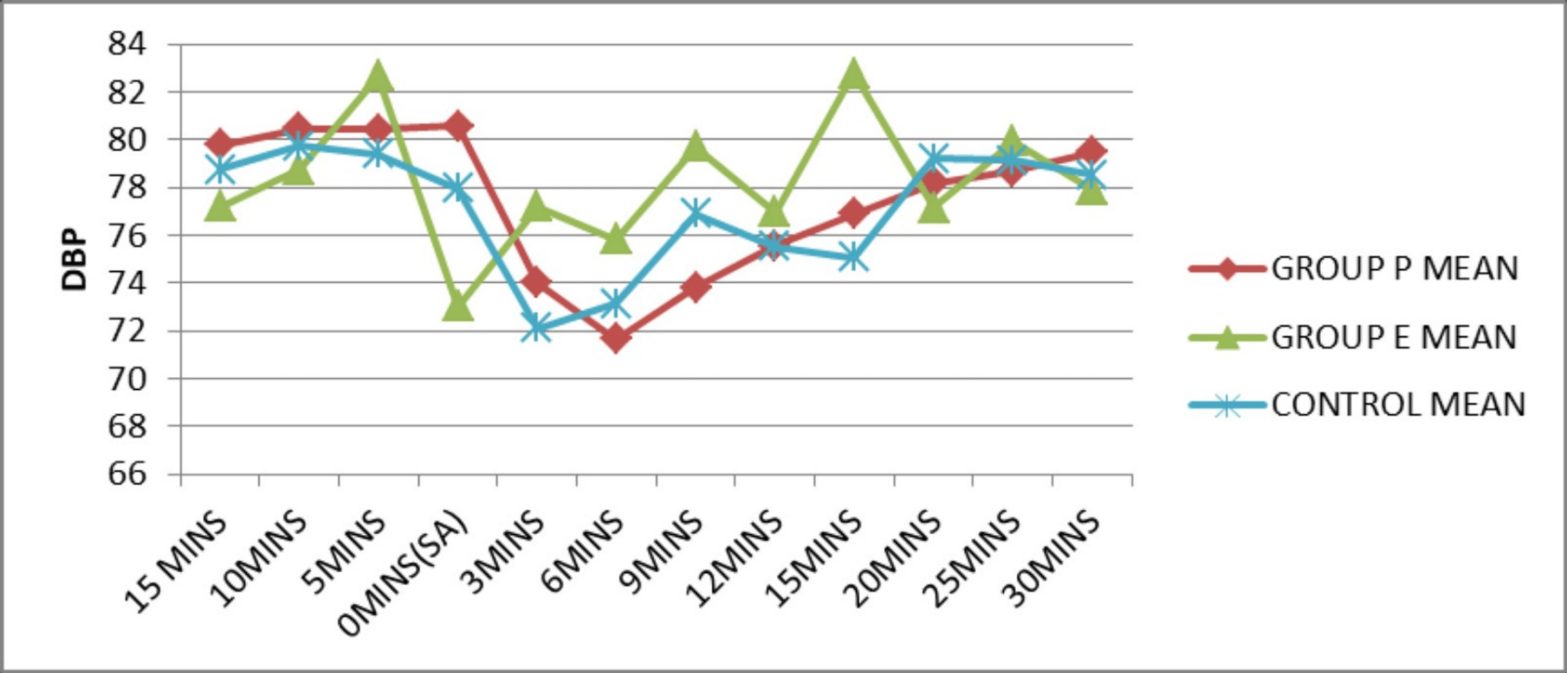
Comparison of DBP before and after SA DBP: Diastolic blood pressure; SA: Spinal anesthesia

The mean diastolic blood pressure at different time intervals was plotted for each group (Figure 3). The mean diastolic blood pressure at induction (0 min) of spinal anesthesia was superior in Group P compared to Group E and the control group. It was enhanced in Group E with reference to other groups at 3 (p=0.002), 6 (p=0.002), 9 (p=0.001), 12 (p=0.02), 15 (p=0.001) and 25 (p=0.01) minute time intervals after spinal anesthesia, which was statistically significant (p<0.05).

**Figure 4:**
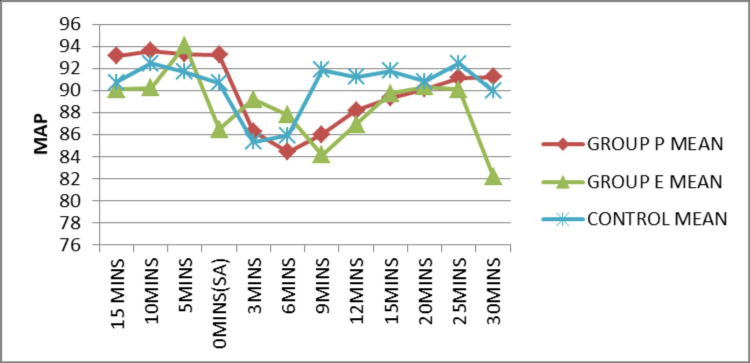
Comparison of MAP before and after SA MAP: Mean arterial pressure; SA: Spinal anesthesia

The mean values of MAP at different time intervals were plotted and the mean MAP was superior in the control group compared to other groups (Figure 4). The mean MAP was enhanced in the ephedrine group in comparison to Group P at 3 (p=0.02), 6 (p=0.017), 15 (0.007), and 20 (0.006) minute time intervals after spinal anesthesia, which was statistically significant (p<0.05).

**Figure 5:**
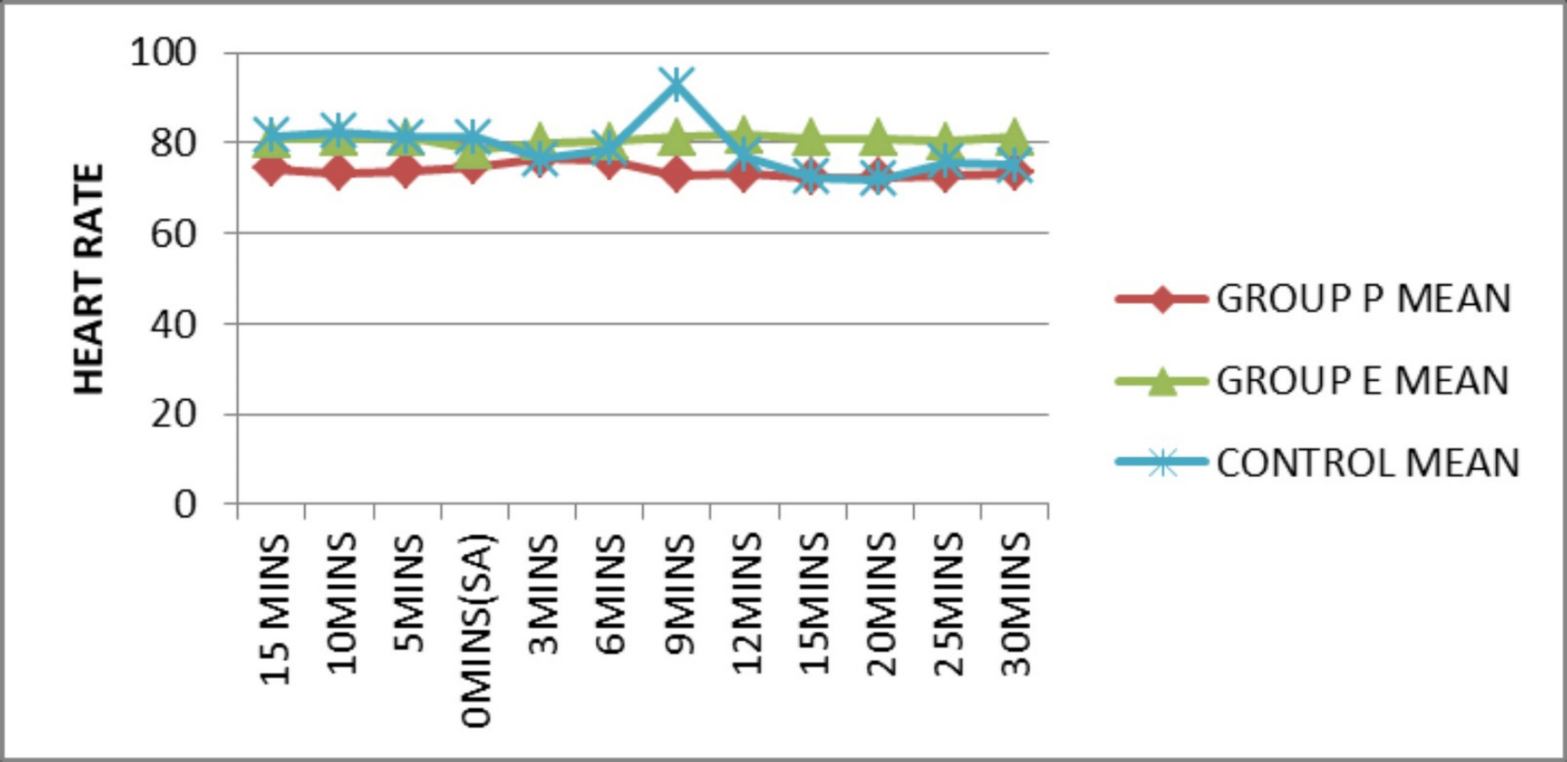
Comparison of heart rate before and after SA SA: Spinal anesthesia

The mean heart rate at different time intervals was plotted for each group (Figure 5). The mean heart rate was superior in Group E in comparison to other groups and was statistically significant (p<0.05) at 3 (p=0.013), 6 (p=0.014), 12 (p=0.001), 15 (p=0.001), 20 (p=0.001), 25 (p=0.001), and 30 (p=0.001) minute time intervals after spinal anesthesia. 

The number and percentage of rescue doses of vasopressors to treat hypotension if it falls below 30% of baseline, given among all groups, were evaluated using the chi-square test and tabulated (Table 1). The obtained p-value of 0.297 was observed to be statistically insignificant.

**Table 1:** Number and percentage of rescue doses given in all groups

Rescue Doses	Group P		Group E		Control		Chi-Square Test	p-Value
	No.	%	No.	%	No.	%		
0	48	82.80%	53	91.40%	45	77.60%	4.911	0.297
1	9	15.50%	5	8.60%	11	19.00%		
2	1	1.70%	0	0.00%	2	3.40%		

The number and percentage of atropine I/V to treat bradycardia (heart rate less than 50 beats/min) given among all groups was tabulated and it was observed to be statistically insignificant (Table 2). The atropine I/V required to treat bradycardia was less in Group E than in Group P and the control group.

**Table 2:** Number and percentage of atropine doses given in all groups

Atropine	Group P		Group E		Control		Chi-Square Test	p-Value
	No.	%	No.	%	No.	%		
0	54	93.10%	56	96.60%	55	94.80%	0.703	0.704
1	4	6.90%	2	3.40%	3	5.20%		

## Discussion

The present study observed the hemodynamic variations after spinal anesthesia. Results showed that the mean systolic blood pressure (Group E>Group P>Group C), diastolic blood pressure (Group E>Group P>Group C), MAP (Group E=Group P=Group C), and heart rate were better maintained in Group E compared to Groups P and C above baseline blood pressure before induction. Elderly patients with untreated sympathetic block after spinal anesthesia have lower systolic blood pressure, systemic vascular resistance, and mean venous pressure due to decompensated state. Restoring preload to the heart during spinal block should be accomplished by administering sufficient intravenous fluids (8-10 ml/kg). Adequate preloading precludes a decrease in cardiac output and unanticipated cardiac arrests, but excessive fluid administration can lead to fluid overload [8]. A vasopressor should be administered if the systolic blood pressure declines by 25 to 30% baseline value or less than 90 mmHg. Vasopressors are known to cause vasoconstriction by increasing systemic vascular resistance to prevent spinal-induced hypotension (SIH) eventually, thus they are a better choice for maintaining hemodynamics in elderly patients [9]. The alpha-adrenergic agonist phenylephrine is typically linked to reflex bradycardia [10]. It raises blood pressure due to venoconstriction and arterial vasoconstriction. It increases venous tone, venous return (preload), and systemic vascular resistance.

Ephedrine, a mixed-action (alpha and beta) adrenergic agonist, increases the cardiac output and prevents a decrease in heart rate as it causes greater venoconstriction than arteriolar constriction and results in increased venous return (preload) and enhances cardiac output, blood pressure, and heart rate [11].

As per the observed results in the present study, phenylephrine 250 mcg/30 ml and ephedrine 30 mg/30 ml, were both effective in maintaining hemodynamic stability after subarachnoid block. The systolic blood pressure was well maintained in Group E>Group P>Group C following induction of spinal block. The attenuation of systolic blood pressure due to sympathetic blockade was more pronounced within the initial 15 minutes after spinal anesthesia and well optimized by Group E compared to Groups P and C.

The diastolic blood pressure was enhanced in Group E>Group P>Group C. The MAP was observed to be equally maintained above and at baseline in all groups (Group P=Group E=Group C). However, the initial drop in MAP following the spinal block was better optimized in the ephedrine group. Zunic et al. reported the impact of phenylephrine or ephedrine infusion following spinal anesthesia on hemodynamics in elderly patients for orthopedic surgeries. They observed that cardiac index, MAP, and heart rate were better in Group E than in Groups P and C [7]. Conversely, Abbasivash et al. found that the MAP, systolic and diastolic blood pressures, and the frequency of hypotension were all considerably reduced in the phenylephrine group after spinal anesthesia, with no apparent difference in heart rate among phenylephrine and ephedrine groups. Less vasopressor use was observed in the phenylephrine group [12]. In this study, less vasopressor rescue dose was required to treat hypotension in Group E compared to Groups P and C. Mon et al. demonstrated that Groups P and E achieved good systolic blood pressure, but ephedrine increased cardiac output in comparison to phenylephrine in parturients [13]. Asokan et al. concluded that ephedrine 6 mg and phenylephrine 100 mcg did not differ in their effectiveness in treating hypotension during subarachnoid block for cesarean section. However, maternal bradycardia was more in the phenylephrine group, with equal incidence of fetal acidosis in the study groups [14]. In this study, both ephedrine and phenylephrine were effective in maintaining hemodynamics and considerably better in the ephedrine group. The incidence of bradycardia was also less in the ephedrine group compared to other groups.

The heart rate in the present study was maintained better in Group E>Group C>Group P. Four patients in the phenylephrine group were given atropine 0.6 mg I/V in response to bradycardia, whereas in the Ephedrine group, only two patients had bradycardia. The rescue doses (phenylephrine 50 mcg/ephedrine 5 mg) to maintain systolic blood pressure after a fall of more than 30% below baseline, were required more in the phenylephrine group than in the ephedrine group. Sinha et al. conducted a study and concluded that following subarachnoid block for cesarean delivery, the three vasopressors efficiently preserved arterial blood pressure. When compared to ephedrine and mephentermine, phenylephrine significantly lowered heart rate [15].

Limitations

A larger sample size is required and a multi-centric study is preferred. Invasive blood pressure (IBP) monitoring provides precise monitoring of blood pressure, which was not done in the present study. Cardiac output is not measured which is crucial in determining the effectiveness of vasopressors among the elderly population.

## Conclusions

Hypotension following spinal anesthesia, caused by a reduction in stroke volume can be treated with crystalloid or colloidal solutions. Administration of vasopressor agents increases stroke volume and preload by increasing systemic vascular resistance without heart stimulation. Hence, both vasopressors (ephedrine and phenylephrine) can be used along with crystalloid solutions to correct hypotension post-subarachnoid block in elderly patients. Consequently, prophylactic low-dose infusion of ephedrine (0.4-0.5 mg/kg) could be a better vasopressor agent of choice than phenylephrine in maintaining hemodynamic stability in elderly patients aged 60 years and above for lower limb orthopedic surgeries under spinal anesthesia.
